# Supplementary material for: The interactome of intact mitochondria by cross-linking mass spectrometry provides evidence for coexisting respiratory supercomplexes
Source: Mol Cell Proteomics. 2017 Dec 8;17(2):216–32. doi: 10.1074/mcp.RA117.000470 (PMC5795388; doi:10.1074/mcp.RA117.000470)
Supplement: Supplemental Data [file supp_RA117.000470_133922_0_supp_23978_fzffwf.zip › spectra_annotation/mito_DR_spectra_annotation/479-1-2-1-4-1.pdf]

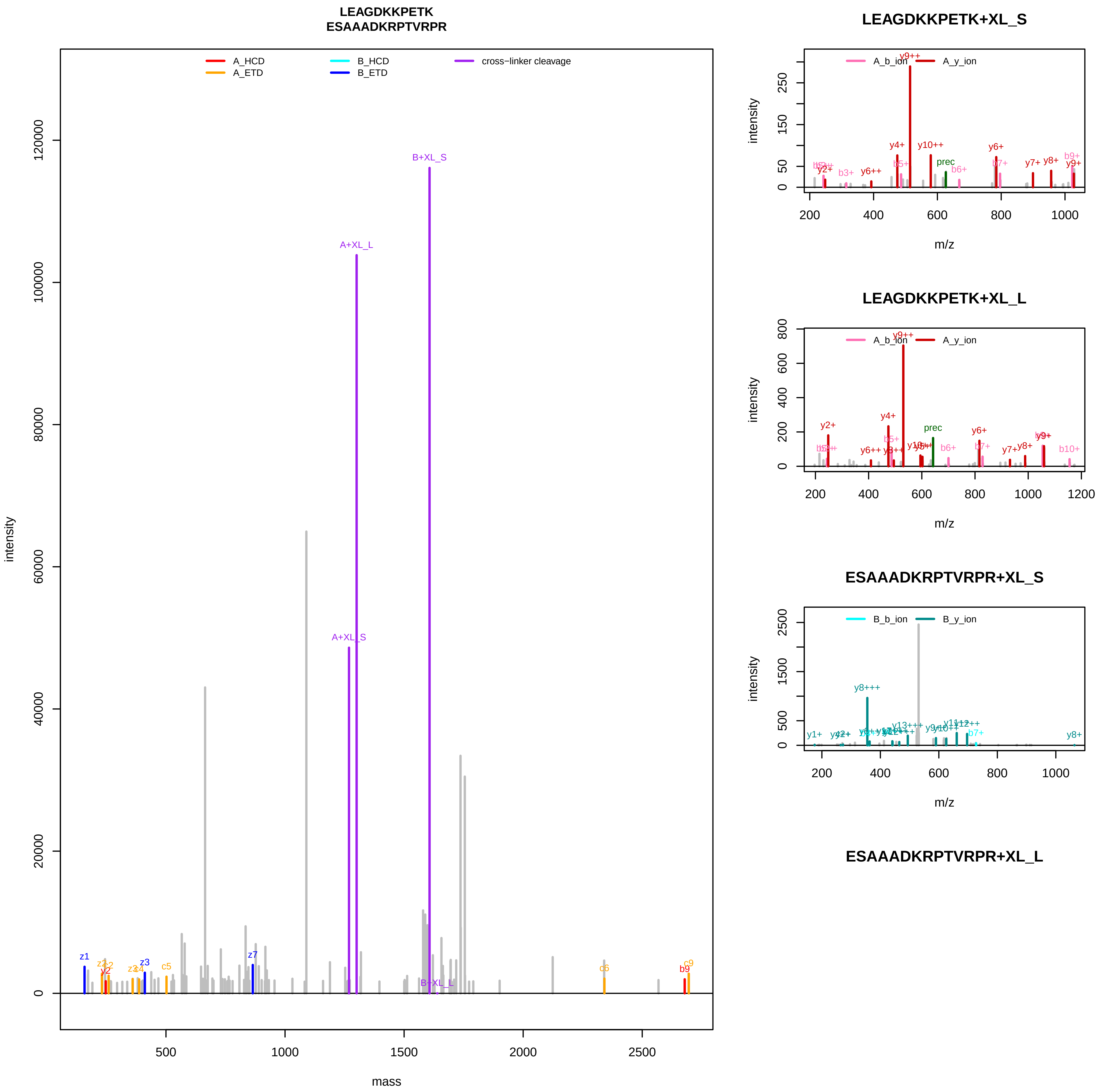

LEAGDKKPETK
ESAAADKRPTVRPR
A_HCD
A_ETD
B_HCD
B_ETD
cross−linker cleavage
B+XL_S
A+XL_L
A+XL_S
B+XL_L
z1
z2
y2
z3
c4
z3
c5
z7
c6
b9
c9
intensity
mass
0
20000
40000
60000
80000
100000
120000
500
1000
1500
2000
2500
LEAGDKKPETK+XL_S
A_b_ion
A_y_ion
y9++
y4+
y10++
y6+
prec
y2+
b3+
y6++
b5+
b6+
b7+
y7+
y8+
b9+
y9+
m/z
LEAGDKKPETK+XL_L
y2+
y4+
y6++
b5+
y10++
prec
b6+
y6+
b7+
y7+
y8+
y9+
b10+
ESAAADKRPTVRPR+XL_S
B_b_ion
B_y_ion
y8+++
y1+
y13+++
y11++
y12++
b7+
y8+
ESAAADKRPTVRPR+XL_L
